# Two new species of *Harpactea* (Araneae, Dysderidae) from Turkey

**DOI:** 10.3897/zookeys.145.1713

**Published:** 2011-11-04

**Authors:** Kadir Boğaç Kunt, Mert Elverici, Recep Sulhi Özkütük, Ersen Aydın Yağmur

**Affiliations:** 1 Poligon Sitesi 71/27-B TR-06810 Dodurga, Çayyolu, Ankara, Turkey; 2 Department of Biological Sciences, Faculty of Arts and Sciences, Middle East Technical University, TR-06531 Ankara, Turkey; 3 Department of Biology, Faculty of Science, Anadolu University, TR- 26470 Eskişehir, Turkey; 4 Ege University, Science Faculty, Biology Department, Zoology Section, TR-35100 İzmir, Turkey

**Keywords:** Dysderidae, Harpacteinae, Eastern Mediterranean, Aegean

## Abstract

Two new species, *Harpactea arnedoi* **sp. n.** and *Harpactea kencei* **sp. n.**, are described on the basis of both sexes from the eastern Mediterranean and Aegean regions of Turkey. *Harpactea kencei* **sp. n.** can be easily distinguished from all other Turkish and European representatives of the genus by the structure of the flattened, massive embolus on the male copulatory organ. Although resembling *Stalagtia* in palpal morphology, we describe one of the new species as *Harpactea arnedoi* **sp. n.** For both Turkish species, detailed morphological descriptions and diagnoses are presented together with figures of the copulatory organs.

## Introduction

The first records of the genus *Harpactea* from Turkey were *Harpactea babori* from İstanbul Province and *Harpactea sturanyi* from Konya Province (Nosek 1905). However, the majority of Turkish *Harpactea* species were described by Brignoli (1978a, b, 1979). In recent years, Lazarov and Deltshev (2008) and Lazarov (2010) re-described *Harpactea sanctaeinsulae* and *Harpactea babori* respectively, which were previously only known from males. Bayram et al. (2009) described *Harpactea christodeltshevi* from the south-eastern Anatolia and Kunt et al. (2010) described *Harpactea erseni*, from the Aegean region. Currently 20 species are known from Turkey (Bayram et al. 2011).

During our surveys of the Turkish spider fauna, we have encountered two new species of *Harpactea* from the eastern Mediterranean and Aegeanregions. The purpose of this study is to describe and diagnose these two new species on the basis of both sexes.

## Materials and methods

**Figure 1.**
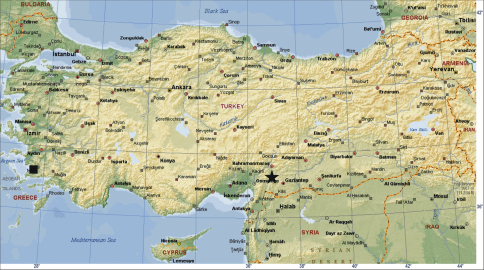
Type localities of the new species. ★ *Harpactea arnedoi*sp. n. ■ *Harpactea kencei* sp. n.

Specimens were collected by means of pitfall traps, sifter and hand collecting from two different provinces of Turkey (Fig. 1). Digital images of the pedipalps and vulvae were taken with a Leica DFC295 digital camera attached to a Leica S8AP0 stereomicroscope, with 5–15 photographs taken in different focal planes and combined using image stacking software. Photographic images were edited using PHOTOSHOP CS2 and COREL-DRAW X3 was used to create the plates. All measurements are in mm. Terminology for the body measurements and copulatory organs follows Chatzaki and Arnedo (2006).

The following abbreviations are used in the text and figures: Carapace and abdomen: AL, abdominal length; CL, carapace length; CWmax, maximum carapace width; CWmin, minimum carapace width. Eyes: AME, anterior median eyes; PLE, posterior lateral eyes; PME, posterior median eyes; AMEd, diameter of anterior median eyes; PLEd, diameter of posterior lateral eyes; PMEd, diameter of posterior median eyes. Chelicera: ChF, length of cheliceral fang; ChG, length of cheliceral groove; ChL, total length of chelicera (lateral external view). Legs: Ta, tarsus; Me, metatarsus, Ti, tibia; Pa, patella; Fe, femur; Tr, trochanter; C, coxa; D, dorsal; Pl, prolateral; Rl, retrolateral; V, ventral. Male bulb: AA, accessory apophysis; Co, conductor; E, embolus. Vulva: aba, anterior basal arc; As, anterior spermatheca; btas, basal transverse part of the anterior spermatheca; dc, distal crest; des, distal expansion of the spermatheca; pd, posterior diverticulum; rsas, rod-shaped part of the anterior spermatheca; tb, transverse bar. Depository: AUZM, Anadolu University, Zoology Museum, Eskişehir, Turkey; cKBK, Personal collection of Kadir Boğaç Kunt, Ankara, Turkey; SMF, Senckenberg Museum, Frankfurt am Main, Germany.

## Taxonomy

**Family Dysderidae C. L. Koch, 1837**

**Genus *Harpactea* Bristowe, 1939**

### Harpactea arnedoi sp. n.

urn:lsid:zoobank.org:act:8BC9D2B7-4E3D-4A17-8017-6DAB08F82D8F

http://species-id.net/wiki/Harpactea_arnedoi

Figs 2 8 

#### Material examined.

**Holotype** ♂ (AUZM), Turkey, Gaziantep Province, Kuşçubeli Pass [37°6'50.20"N; 36°36'34.20"E], 13.XI.2010, under leaf litter, leg. E.A.Yağmur. **Paratypes** 1 ♀, 1 ♂ (AUZM); 2 ♀♀, 2 ♂♂, 9 juveniles (cKBK & SMF), same data as holotype.

#### Derivatio nominis.

The new species is dedicated to the Spanish arachnologist, Dr. Miquel Arnedo (Barcelona University, Barcelona, Spain), who has made important contributions to the taxonomy of the family Dysderidae.

#### Diagnosis.

*Harpactea arnedoi* sp. n. differs from all other *Harpactea* species in the structure of the copulatory organs. However, the general morphology of the male palp resembles that of *Harpactea zoiai* Gasparo, 1999 known from Greece (see Gasparo 1999). The male of *Harpactea arnedoi* sp. n. differs in having an uneven spherical shape of the palpal bulb; the region between the bulb and the distal continuation is elongated and has a funnel-like appearance; the embolus is shorter, hook-like and bends downwards towards the tip. The vulva is apparent and characterized by a peripherally sclerotized posterior diverticulumand folded distal expansion.

**Figures 2–6.**
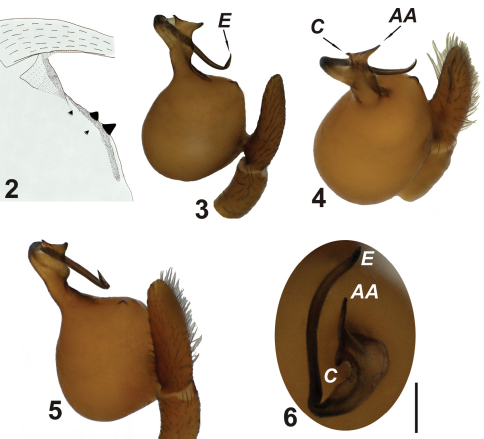
*Harpactea arnedoi* sp. n. **2** Cheliceral teeth **3** left palp, retrolateral view **4** ditto, nearly anterior view **5** ditto, nearly retrolateral view **6** ditto, distal view. Scale line: 0.5 mm.

**Figures 7–8.**
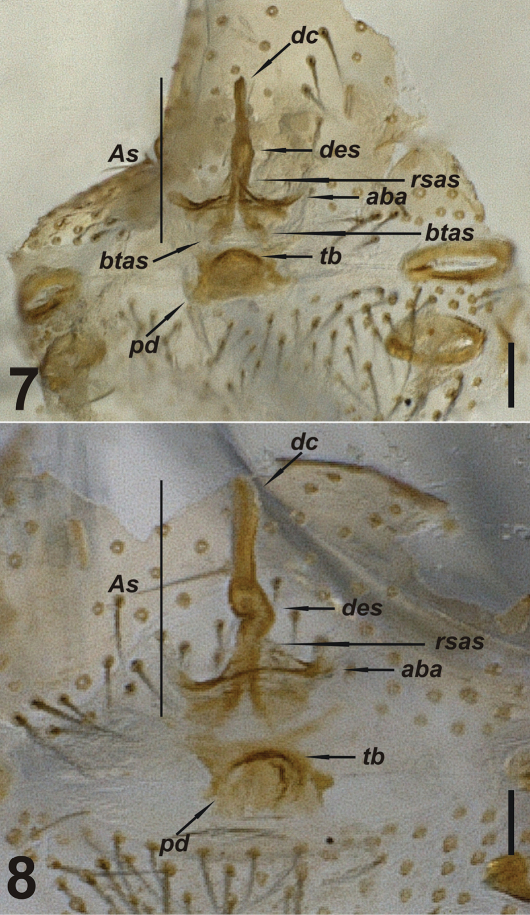
Vulva of *Harpactea arnedoi* sp. n. **7** dorsal view **8** ventral view. Scale lines: 0.1 mm.

#### Notes.

While describing *Harpactea arnedoi* sp. n., it was difficult to decide whether it would be best assigned to *Harpactea* or to *Stalagtia*. By the characteristic structure of the male copulatory organ (globular tegulum, long embolus, poorly developed conductor), the new species is similar to members of *Stalagtia*. However, the male palp possesses a poorly developed conductor (Fig. 4), and the female copulatory organs are similar to those of *Harpactea* species in having a short posterior diverticulum and anterior spermathecae. Thus, we feel the new species is correctly placed in *Harpactea*. Further evidence in support of this generic placement is the absence of ventral spines on the anterior tibiae and metatarsi in the new species, particularly as Deeleman-Reinhold (1993) stated that their presence was diagnostic of *Stalagtia*. However, the only known Turkish species of *Stalagtia*, *Stalagtia thaleriana* Chatzaki & Arnedo, 2006, does not possess ventral spines on the anterior tibiae and metatarsi, as was mentioned earlier by Chatzaki and Arnedo (2006) and Kunt et al. (2009).

#### Description.

Measurements: [Holotype ♂ / Paratype ♀]: AL 2.26 / 2.50; CL 1.75 / 1.73; CWmax 1.45 / 1.35; CWmin 0.63 / 0.65; AMEd 0.10 / 0.11; PLEd 0.09 / 0.09; PMEd 0.08 / 0.08; ChF 0.37 / 0.38; ChG 0.18 / 0.19; ChL 0.62 / 0.64 mm. Leg measurements are given in Table 1.

**Table 1.** Leg measurements of *Harpactea arnedoi* sp. n.<br/>

**(Holotype ♂ / Paratype ♀)**	**Fe**	**Pa**	**Ti**	**Me**	**Ta**
**Leg I**	1.42 / 1.23	0.80 / 0.73	1.15 / 0.98	1.05 / 0.78	0.38 / 0.32
**Leg II**	1.10 / 1.17	0.80 / 0.75	1.12 / 0.93	1.05 / 0.85	0.37 / 0.31
**Leg III**	1.05 / 1.00	0.55 / 0.55	0.83 / 0.63	1.02 / 0.95	0.37 / 0.32
**Leg IV**	1.63 / 1.50	0.83 / 0.78	1.40 / 1.25	1.50 / 1.35	0.40 / 0.33

MALE: Small sized spider. Carapace greenish - light brown, with smooth surface and distinct fovea. AME, PLE and PME closely grouped; AME separated. Sternum, labium, gnathocoxae and chelicerae light brown. Sternum dark brown laterally, with thin, long hair near margins. Cheliceral groove with four teeth: retromargin with two tiny teeth; promargin with two strong teeth, largest tooth closest to base of the cheliceral groove (Fig. 2). Top of the labium and gnathocoxae with short, strong hair, sparsely distributed. Abdomen greyish-light brown, with short, thin blackish hair over the entire surface. Legs yellowish-light brown with sparse blackish setae. Ventral surface of coxae with long, thin, black sparse hair. Leg IV > Leg I > Leg II > Leg III. Tarsi with three claws. Tarsi III and IV with fine scopulae. Legs III and IV with fine metatarsal scopulae covering slightly less than the distal half of the segment (ventral surface only). Prolateral part of coxae III and IV with 1 spine. Dorsal parts of femora, tibiae and metatarsi with spines. Number of spines can vary among individuals. Detailed leg spination of *Harpactea arnedoi*sp. n. is given in Table 2.

**Table 2.** Leg spination of *Harpactea arnedoi*sp. n.<br/>

♂ **(Holotype)**	**Leg I**	**Leg II**	**Leg III**	**Leg IV**
**C**	0	0	1 Pl	1 Pl
**Fe**	2 Pl	1-2 Pl	1, 1 Pl 1, 1, 1 D	2 Pl 1 D
**Pa**	0	0	1 Rl	0
**Ti**	0	0	1, 1 Pl 2, 1, 1 Rl 1, 1, 2 V	1, 1 Pl 1 D 2, 1, 1 Rl 1, 1, 2 V
**Me**	0	0	1, 1 Pl 1, 1, 1 Rl 1, 1, 2 V	1, 1, 1 Pl 1, 1, 1 D 4 Rl 1, 1, 2 V
♀ **(Paratype)**				
**C**	0	0	1 Pl	1 Pl
**Fe**	2 Pl	1 Pl	1 Pl 1, 1 D	2 Pl 2 D
**Pa**	0	0	1 Rl	0
**Ti**	0	0	1, 1 Pl 2, 1, 1 Rl 1, 1, 2 V	1, 1, 1 Pl 1 D 2, 1, 1 Rl 1, 1, 2 V
**Me**	0	0	1, 1, 1 Pl 1, 1 Rl 1, 1, 2 V	1, 1, 2 Pl 1, 1 D 4 Rl 1, 1, 2 V

Palpal tarsus of the male covered with thin and elongated setae. Tegulum yellow, lighter than the legs. Bulb almost spherical. The anteroventral region of the bulb has a chitinized edge. Between bulb and distal appendages there is a neck-shaped transition region (Figs 3–5). This region is lightly chitinized and dusky in patches. From the base of the embolus, conductor and accessory apophysis have dark brown tips. Embolus is slender and cylindrical up to its hook-like tip and it is not so heavily sclerotized. At the periphery it is membranous and projects downwards, parallel to the palpal tibia. Conductor and accessory apophysis are developed at the end of the neck-shaped transition region, on the opposite tips of an ear-shaped structure. Both are triangular in shape, whereas the accessory apophysis is longer and jagged (Fig. 6). The relative positions of the distal appendages are most easily seen at a 90 degree angle in ventral view. Embolus separated from conductor and accessory apophysis by a broad base, at first it follows the course of the conductor, but later bends with a sharp curve and accompanies the accessory apophysis.

FEMALE: No differences found between male and female, in terms of body colour and morphology. Vulva sclerotized almost uniformly. Distal crest long and butt-ended at the tip. Distal expansion of spermatheca convoluted. Rod-shaped part of the anterior spermatheca short and cylindrical. Basal transverse part of the anterior spermatheca separates from rod-shaped part laterally at an acute angle and forms a triangular shape. Anterior basal arc lies linearly through the centre and periphery, but widens at the edges. Transverse bar short and crescent-shaped with tips turned downwards. Posterior diverticulum shaped as a membranous sac and is sclerotized at the periphery (Figs 7, 8).

#### Ecology.

Specimens were collected in Kuşçubeli Pass, located in the Amanos Mountains, from habitats covered by Turkish Pine (*Pinus brutia*) and scrub type oak forests (*Quercus infectoria*). A variety of herbaceous plants and low shrubs such as *Ruscus aculeatus* are also widely represented in those forests. Sampling was done by sifting leaf litter during the early winter.

#### Distribution.

*Harpactea arnedoi*sp. n. is known from thetype locality only.

### Harpactea kencei sp. n.

urn:lsid:zoobank.org:act:557FE296-4BF3-4E21-A8D6-4750C38795E6

http://species-id.net/wiki/Harpactea_kencei

Figs 9 15 

#### Material examined.

**Holotype** ♂ (AUZM), Turkey, Muğla Province, Milas District, Kıyıkışlacık Village [37°16'38.80"N; 27°33'47.97"E], 13.XI.2010, under stones, leg. M.Elverici. **Paratypes** 1 ♀ (AUZM), 1 ♀ 5 ♂♂ (cKBK & SMF), same data as holotype.

#### Derivatio nominis.

The new species is named in honor of the well-known Turkish biologist Prof. Dr. Aykut Kence (Middle East Technical University, Ankara, Turkey) for his important contributions to Turkish biology.

#### Diagnosis.

*Harpactea kencei* sp. n. can be easily distinguished from all other Turkish *Harpactea* by the unique structure of the male palp and broad, grooved sides, crescent like anterior basal arc-shaped structure of the female vulva. In general appearance the embolus is well sclerotized, thorn-like and with a hook-shaped tip. Towards the tip, the embolus flattens like a spoon and ends with a thorn-like tip inflecting upon itself. On the embolar base there is a small ear-shaped conductor relatively well sclerotized at its peripheries. This is attached to the embolus at an angle of 90 degrees. Unique embolus (not a simple thornlike structure) and the presence of the conductor differentiate *Harpactea kencei* sp. n. from *Harpactea diraoi*, *Harpactea isaurica*, *Harpactea sanctaeinsulae* and any other similar Turkish species, which all have simple spiniform embolus structures (see Brignoli 1978b).

#### Description.

Measurements: **[Holotype** ♂ **/ Paratype** ♀**]:** **AL** 2.13 / 2.25; **CL** 1.63 / 1.96; **CWmax** 1.35 / 1.53; **CWmin** 0.63 / 0.75; **AMEd** 0.07 / 0.08; **PLEd** 0.05 / 0.07; **PMEd** 0.05 / 0.06; **ChF** 0.34 / 0.37; **ChG** 0.21 / 0.27; **ChL** 0.64 / 0.71 mm. Leg measurements are given in Table 3.

**Figures 9–13.**
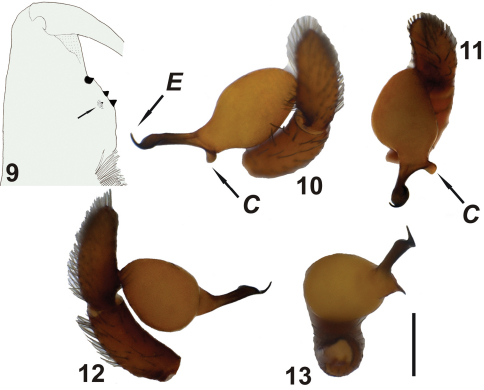
*Harpactea kencei* sp. n. **9** Cheliceral teeth **10** left palp, retrolateral view **11** ditto, nearly anterior view **12** ditto, prolateral view **13** ditto, distal view. Scale line: 0.5 mm.

MALE: Small to medium sized spider. Carapace reddish dark brown, with smooth surface and distinct fovea. AME, PLE and PME closely grouped; AME separated. Difference between width of eyes region and thoracic region of carapace remarkable. Sternum, labium, gnathocoxae and chelicerae reddish-brown. Sternum with long, thin hair near the margin, while centrally smooth and shiny. Cheliceral groove with four teeth: retromargin bears a tiny tooth at the base of the groove and a strong tooth at the top; promargin with two strong teeth of almost equal sizes (Fig. 9). Labium with short, strong hair, sparsely distributed along the surface; hair cover denser at the top. Gnathocoxae also with moderately strong hair, denser and slightly longer at the margins. Abdomen yellowish-light brown, with short, thin blackish hair over the entire surface. Legs yellowish-light brown with sparse blackish setae. Anterior legs slightly darker than posterior legs. Leg IV > Leg I > Leg II > Leg III. Tarsi with three claws. Tarsi III and IV with fine scopulae. Legs III and IV with fine metatarsal scopulae covering slightly less than the distal half of the segment (ventral surface only). Prolateral part of coxae III and IV with 0-5 spines. Detailed leg spination of *Harpactea kencei* sp. n. is given in Table 4.

**Table 3.** Leg measurements of *Harpactea kencei* sp. n.

**(Holotype** ♂ **/ Paratype** ♀**)**	**Fe**	**Pa**	**Ti**	**Me**	**Ta**
**Leg I**	1.57 / 1.61	0.95 / 0.96	1.28 / 1.29	1.25 / 1.27	0.48 / 0.48
**Leg II**	1.48 / 1.50	0.80 / 0.82	1.23 / 1.25	1.20 / 1.22	0.40 / 0.40
**Leg III**	1.13 / 1.17	0.56 / 0.63	0.80 / 0.88	0.90 / 1.13	0.40 / 0.40
**Leg IV**	1.62 / 1.63	0.82 / 0.87	1.45 / 1.57	1.52 / 1.75	0.51 / 0.53

**Table 4.** Leg spination of *Harpactea kencei* sp. n.<br/>

♂ **(Holotype)**	**Leg I**	**Leg II**	**Leg III**	**Leg IV**
**C**	0	0	1Pl	5 Pl
**Fe**	2, 1 Pl	1, 1, 1 Pl	1, 1 D 1, 1 Rl	3, 1, 1 D
**Pa**	0	0	1 Rl	0
**Ti**	0	0	1, 2 Pl 2, 1, 2 Rl 1, 1, 2 V	2, 1, 1 Pl 2, 1, 1 Rl l 1, 1, 2 V
**Me**	0	0	1, 1, 1 Pl 5 Rl 2 V	1, 1, 1 Pl 1, 1, 1 Rl 3, 2 V
♀ **(Paratype)**				
**C**	0	0	0	1 Pl
**Fe**	2 Pl	2 Pl	1, 1 D 1, 1 Rl	2, 1, 1 D
**Pa**	0	0	1 Rl	0
**Ti**	0	0	1, 1 Pl 2, 1, 1 Rl 1, 1, 2 V	2, 1, 1 Pl 2, 1, 1 Rl 1, 1, 2 V
**Me**	0	0	1, 1 Pl 6 Rl 2, 2 V	1, 1, 1 Pl 1, 1, 1 Rl 2, 1, 2 V

Tegulum yellowish brown, longer than wide, cylindrical. Embolar base broad, embolus and conductor reciprocally located at peripherals. Accessory apophysis absent. Embolus blackish brown, flattens like a spoon through the tip and ends with a thorn-like tip inflecting upon itself. Conductor small, flattened and ear-shaped, separated from embolus at the base.

**Figures 14–15.**
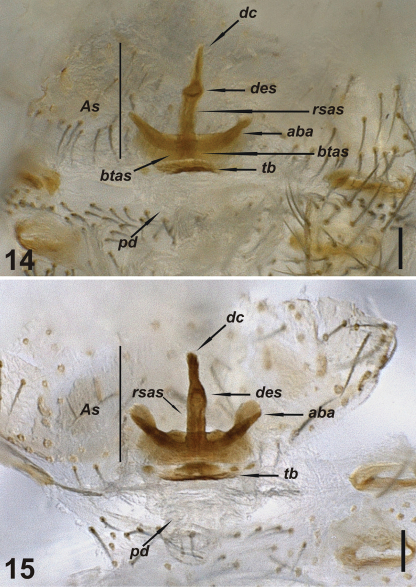
Vulva of *Harpactea kencei* sp. n. **14** dorsal view **15** ventral view. Scale lines: 0.1 mm.

FEMALE: No differences found between male and female, in terms of body colour and morphology. Distal crest of vulva short, with conical tip. Distal expansion of spermatheca spherical, with triangular shape. Basal transverse part of the anterior spermatheca short, linear and peripherally sclerotized. Rod-shaped part of the anterior spermatheca elongated. Anterior basal arc crescent-like and grooved. Tips membranous, in the shape of half of a heart. Transverse bar smooth, well sclerotized at the center. Posterior diverticulum membranous (Figs 14–15).

#### Ecology.

Specimens were collected in the course of field studies aiming to determine the spider fauna of olive groves found in Kıyıkışlacık Village. The area was surveyed periodically over one year using pitfall traps, sweep nets, active searching at ground level and under stones both during the day and at night, sifting tree litter and by shaking tree branches. The altitude of the study area varied from sea level up to 100 m at its maximum.

Individuals of the new species were found under stones and collected from pitfall traps in olive groves and shrub forest associations dominated by *Quercus* trees. The first adult specimen was collected in November and additional specimens were found during March and April. It is likely that this species is most active during the early spring.

#### Distribution.

*Harpactea kencei* sp. n. is known from thetype locality only.

#### Comments.

According to the classification established by Deeleman-Reinhold (1993), *Harpactea arnedoi* sp. n. and *Harpactea kencei* sp. n. can be placed within the species group *rubicunda* (D) because they possess the following characteristics:

1.	Posterior diverticulum of the female vulva membranous in both species.

2.	Embolus of *Harpactea arnedoi* sp. n. is spiniform, while it is massive-spiniform and flattened in *Harpactea kencei* sp. n.

3.	Conductor massive in both species.

4.	Patellae and coxae with spines in both species.

## Discussion

The genus *Harpactea* is represented in Turkey by 20 described species. Of these, *Harpactea agnolettii*, *Harpactea colchidis*, *Harpactea galatica*, *Harpactea lazonum*, *Harpactea lyciae*, *Harpactea medeae*, *Herpactes pisidica*, *Harpactea sbordonii* and *Harpactea vignai* were described on the basis of females only; while *Harpactea christodeltshevi*,*Harpactea erseni* and *Harpactea korgei* were described on the basis of males. Due to the inadequacy of the existing descriptions, and the unavailability of many of the type specimens for further investigations, it is currently not possible to draw accurate conclusions about the status of the genus *Harpactea* in Turkey. Nevertheless, the relevant literature shows that most of the recorded species belong in the species group *rubicunda* (D)and*Harpactea* species belonging to this group have often been described from Thrace, Aegean and the Mediterranean coast (see Brignoli 1978a, b, 1979; Lazarov and Deltshev 2008; Lazarov 2010).

With the descriptions of *Harpactea arnedoi* sp. n. and *Harpactea kencei* sp. n. presented herein, the total number of *Harpactea* species reported from Turkeyis now 22. With the exception of *Harpactea babori*, *Harpactea mithridatis* and *Harpactea sturanyi*, all other species are only known only from their type localities or proximate vicinities. *Harpactea babori*, with the type locality in Büyükada (Insel Prinkipo), has a distribution pattern ranging from Belgrad Forest (Silva Belgradensis) which is one of the last remaining relict forests in İstanbul, and lies along the Istranca Mountain range (both Bulgarian and Turkish sides). As the predominant harpacteid spider species of the Istranca Mountains, *Harpactea babori* has been recorded from Shumen Town in Bulgaria as the northernmost record and from Stara Planina as the westernmost record of its known distribution range. *Harpactea mithridatis* has been recorded from Ordu province (East Black Sea Region) in Turkey and from Adzharia region(Khulo) in SW Georgia; while *Harpactea sturanyi*, in addition to its type locality in Konya (Central Anatolia), has also been recorded from the Aegean coast of Turkey (Denizli, İzmir and Muğla provinces; senior author, pers. obs.) and from the Bulgarian side of the Istranca Mountain (see Drensky 1936, 1938). What we would like to emphasize here is the fact that the native *Harpactea* species, which were also recorded from other countries, are principally distributed through zones with similar ecological conditions. Moreover, it is a known fact that other than some exceptional species like *Harpactea hombergi*, *Harpactea lepida* and *Harpactea rubicunda*, species in the genus *Harpactea* show narrow distribution patterns in general. Furthermore, the geological, floristic and climatic conditions of Turkey appear to have been conducive to creating opportunities for isolation and diversification of species within the genus *Harpactea*. With our ongoing research on the spider fauna of Turkey, we expect there will be many more additions to the genus in the near future.

Chatzaki and Arnedo (2006) expressed the taxonomic situation of the genus *Harpactea* in their remarkable revision on the epigean harpacteid spiders of Crete with such words: “*Harpactea* is one of the most ill-defined genera in the whole family and a major taxonomic revision of this genus is urgently called for”. We agree with this and believe that the *Harpactea* fauna of Turkey will play a key role in any future revision of the genus because Anatolia constitutes a transition region between Europe, the Balkans, and the Caucasus and the Middle East.

## Supplementary Material

XML Treatment for Harpactea arnedoi

XML Treatment for Harpactea kencei
